# Induction Implications: Shaping the Competence and Confidence of Junior Doctors Within Complex Medical Specialties

**DOI:** 10.7759/cureus.50768

**Published:** 2023-12-19

**Authors:** Tareq Al Saoudi, Kanika Chawla, Farah Khasawneh, Cristina Pollard, John Isherwood, Neil Bhardwaj, Giuseppe Garcea, Ashley Dennison

**Affiliations:** 1 Hepato-Pancreato-Biliary (HPB) Surgery Department, University Hospitals of Leicester NHS Trust, Leicester, GBR; 2 Colorectal Surgery Department, University Hospitals of Leicester NHS Trust, Leicester, GBR

**Keywords:** quality improvement projects, induction training, foundation doctors, post grad medical education, hpb surgery

## Abstract

Introduction

The newly qualified junior doctors in the United Kingdom face challenges due to their limited experience and unfamiliarity with their rotations. We aim to share the experience of establishing a hepato-pancreato-biliary (HPB) surgery-specific induction program at the University Hospitals of Leicester NHS Trust and assess its impact on doctors' knowledge and experience.

Methods

A booklet was distributed to new junior doctors, and a two-hour structured teaching session was also conducted, with pre- and post-session assessments using multiple-choice questions and a feedback survey. The survey measured understanding of HPB anatomy, interventions, and satisfaction with the teaching methodology.

Results

The pre-session questionnaire included 22 participants, while the post-session had 20 participants. Regarding HPB anatomy understanding, in the pre-session, six (28.6%) and 11 (52.4%) participants reported levels 2 and 3, respectively, while levels 4 and 5 were reported by three (13.3%) and one (4.8%) participants. In the post-session, levels 4 and 5 were reported by six (30%) and 13 (65%), with only one (5%) reporting level 3 and none at levels 1 or 2. Similar trends were observed in understanding HPB investigation. In the pre-session, levels 2 and 3 were reported by eight (36.4%) and 11 (50%), while levels 4 and 5 were reported by two (9.1%) and one (4%). In the post-session, eight (40%) and 11 (55%) reported levels 4 and 5, with only one (5%) at level 3 and none at levels 1 or 2. For HPB management methods before teaching, levels 2 and 3 were equally reported by eight (36.4%), level 4 by four (22.7%), and none at level 5. After teaching, nine (45%) and 10 (50%) reported levels 4 and 5, with only one (5%) at level 3 and none at levels 1 or 2. Factual knowledge showed a 38% increase, rising from 49% pre-session to 87% post-session. In post-session feedback, 12 (60%) strongly agreed that the session helped augment their medical practice, and six (30%) agreed, with two (10%) neutral. Feedback on the teaching session's organization was positive, with 13 (65%) strongly agreeing that it was structured coherently, and six (30%) agreeing, with only one (5%) neutral regarding the clarity of the structure and delivery method.

Conclusion

Specialty-specific induction programs are crucial for providing support and ensuring the development of competent doctors. Efforts should be made to create supportive working environments for junior doctors to alleviate stress and improve their well-being.

## Introduction

The National Healthcare System (NHS) in the United Kingdom has been regarded by many as one of the best in the developed world [1]. Although it is composed of myriad complex organizations and commissioning bodies, doctors remain one of the main pillars of care provision and support for patients [2]. Medical student training prior to qualification is lengthy and mandates exposure to the wide variety of specialties required to safely practice following registration. Newly qualified trainees are required to assimilate a wide number of skills in different areas during their attachment to medical firms and in secondary care, often as part of rotations designed to provide an eclectic mix of experiences [3]. In addition, the last two decades have witnessed significant and wide-ranging changes to the firm structure, which have affected continuity of care and loosened the personal relationships that were inherent in the previous structure.

One of the challenges that newly qualified junior doctors face is the lack of experience at the beginning of a rotation, which is a particular problem in situations where they are expected to work independently, frequently in complex areas [4]. Although many hospitals in the United Kingdom offer structured orientation programs for new junior doctors, which aim to familiarize them with the hospital they are working in, build relationships with more experienced colleagues, and introduce them to the firm in which they will be working [5], there is a paucity of structured induction programs tailored to particular specialties.

The aim of this article was to share our experience in the Hepato-Pancreato-Biliary (HPB) surgery unit at the University Hospitals of Leicester NHS Trust in establishing an HPB surgery-specific induction program, and its effect on the newly joining doctors’ knowledge, experience, and enjoyment of their time on the unit.

## Materials and methods

Survey design and evaluation

The survey used in this study was meticulously designed to assess the effectiveness of the educational interventions provided to junior doctors in the HPB department at the University Hospitals of Leicester. To ensure the survey's content validity and relevance to the targeted medical education objectives, it underwent a rigorous evaluation process by a group of experienced consultants with backgrounds in medical education and expertise in HPB pathologies. These consultants played a vital role in refining and finalizing the survey questions and format to align with the learning goals of the quality improvement project.

Educational interventions

This study was conducted as part of a comprehensive quality improvement project within the HPB department. The project aimed to enhance the knowledge and clinical skills of foundation year one and two doctors who were newly joining the department. The project comprised two main educational components.

Supplemental Booklet

A comprehensive booklet was created and designed by an HPB consultant and a Cancer Nurse Specialist. This booklet contained detailed explanations about various HPB pathologies, commonly employed investigative techniques, and guidelines for performing essential tasks and responsibilities. Additionally, it provided contact details for key departmental resources, including the Interventional Radiology Department, Endoscopy Services, senior medical staff, and cancer nurse specialists. The booklet was distributed to junior doctors during their shadowing period, serving as an essential reference guide.

Structured Teaching Session

A two-hour structured teaching session was organized for junior doctors to cover key HPB topics in a concise and focused manner. The session was designed to be interactive, encouraging active participation, questions, and discussions. Both pre- and post-session assessments were conducted to evaluate the effectiveness of this teaching approach.

Assessment tools

To assess the effectiveness of the educational interventions, two assessment tools were employed.

Pre- and Post-Session Assessment

Before and immediately after the teaching session, junior doctors were given a series of multiple-choice questions. This assessment was designed to measure the knowledge they had gained during the teaching session, and it also included four knowledge-based questions to evaluate their ability to safely carry out tasks related to HPB pathologies.

Feedback Survey

A comprehensive feedback survey was used to measure the junior doctors' understanding of HPB anatomy, investigative procedures, and interventions. The survey utilized a rating scale ranging from 1 to 5, with 5 indicating a high level of understanding and 1 indicating a low level of understanding.

Data collection and analysis

Both the pre- and post-session assessments and the feedback survey were administered using Google Forms to facilitate data collection. The collected data were subsequently analyzed to assess the effectiveness of the teaching session, identify areas for improvement, and measure the increase in knowledge and confidence among junior doctors when dealing with HPB patients. The survey results were analyzed quantitatively, and findings were reported.

## Results

**Figure 1:**
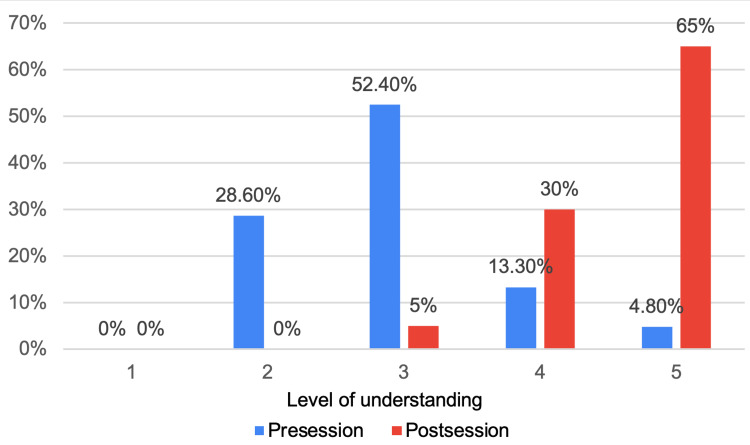
Level of understanding of hepato-pancreato-biliary (HPB) anatomy

The pre-session questionnaire was completed by 22 participants, while the post-session questionnaire was completed by 20 participants. In the initial pre-teaching session questionnaire, we assessed the participants' comprehension of HPB anatomy. The majority of respondents indicated an understanding at levels 2 and 3, with six (28.6%) and 12 (52.4%) participants, respectively. In contrast, only three (13.3%) and one (4.8%) participants reported levels 4 and 5, respectively. However, following the teaching session, there was a notable surge in the participants' grasp of HPB anatomy. Specifically, six (30%) and 13 (65%) participants reported achieving levels 4 and 5, respectively. Significantly, only one participant (5%) reported level 3, and none of the participants indicated levels 1 or 2 in their post-teaching session feedback (Figure 1).

**Figure 2:**
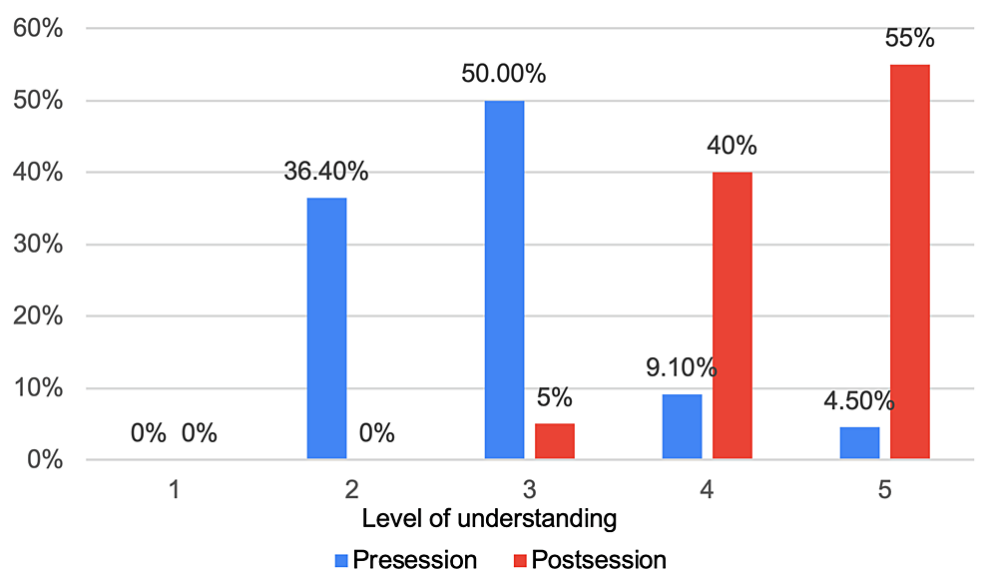
Level of understanding of terms and investigations in hepato-pancreato-biliary (HPB) management

Similar results were observed across the other tested categories. Prior to the teaching session, the majority of junior doctors reported having level 2 and 3 understanding of medical terms and investigations, with eight (36.4%) and 11 (50%), respectively (Figure 2). Level 4 understanding was reported by two participants (9.1%), while only one (4.5%) demonstrated level 5 understanding. Following the teaching session, there was a significant improvement in their understanding, with eight (40%) reporting level 4 and 11 (55%) reporting level 5. Similar to the improvement in the knowledge of HPB anatomy, only one participant (5%) reported level 3, and none of the participants reported levels 1 or 2 (Figure 2).

**Figure 3:**
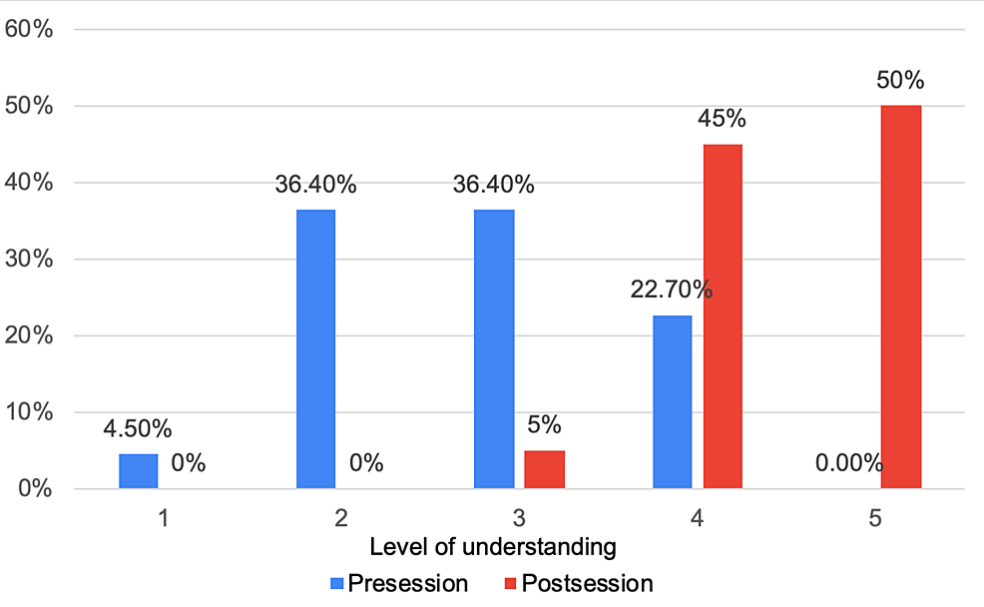
Understanding of management methods in hepato-pancreato-biliary (HPB) management

For questions related to the management methods for HPB diseases, prior to the teaching session, level 1 of understanding was reported by one (4.5%) participant, level 2 and level 3 understanding were equally reported by eight participants (36.4%) each, with level 4 understanding reported by five participants (22.7%), and no participants demonstrating level 5 understanding. However, the teaching session produced a significant improvement, with nine (45%) reporting level 4 and 10 (50%) reporting level 5. Only one participant (5%) reported level 3, and none of the participants reported levels 1 or 2 (Figure 3).

**Figure 4:**
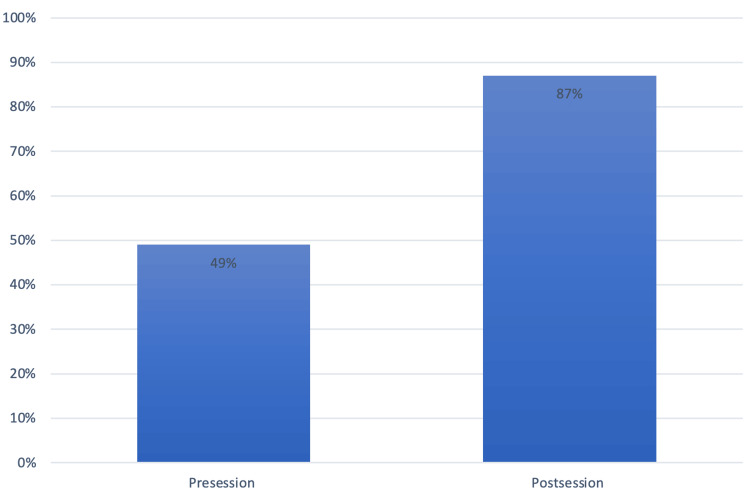
Percentage of correctly answered multiple-choice questions

Overall, there was a significant improvement in factual knowledge among the junior doctors who participated. On average, there was a 38% increase in correct responses to questions that tested basic HPB and surgical knowledge. The pre-teaching questionnaire, completed by 22 junior doctors, revealed that the total average of correct answers was only 49%, compared to the post-teaching questionnaire, where 87% of the answers were correct (Figure 4).

**Figure 5:**
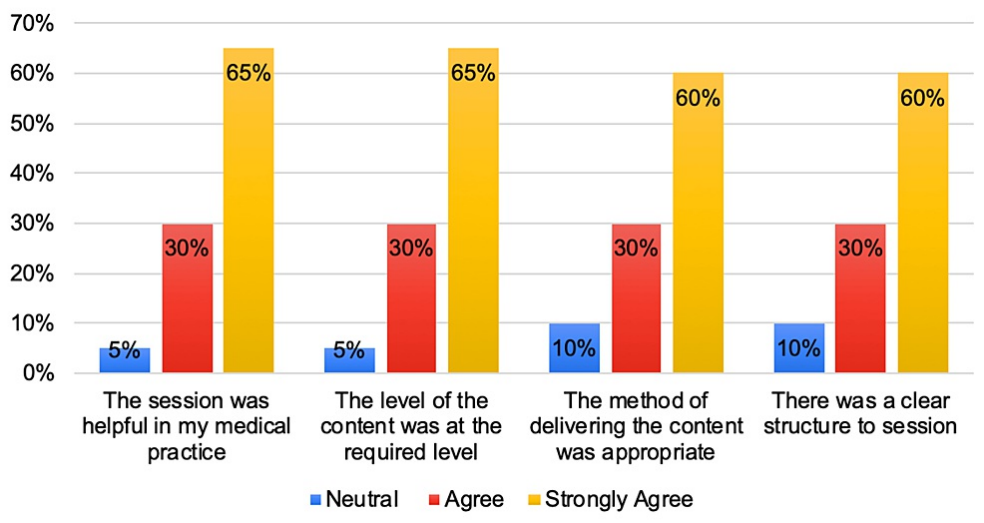
Feedback from the participants

Following the teaching session, the post-session questionnaire was used to gather general feedback from the participants. Twelve participants (60%) of the cohort strongly agreed that the session was helpful in augmenting their medical practice and that the content was presented at an appropriate level. Six participants (30%) expressed agreement, with only two participants (10%) neither agreeing nor disagreeing with the statement. Feedback relating to the organization of the teaching session was also supportive and encouraging, with 13 participants (65%) of respondents strongly agreeing that the session was structured coherently, and that the delivery method was appropriate for their learning requirements. An additional six participants (30%) agreed with these views, with only one participant (5%) neither agreeing nor disagreeing regarding the clarity of the structure and delivery method (Figure 5).

## Discussion

“Changeover days” can be a difficult time for junior doctors who often transition from one rotation or assignment to another, which is substantially different, with only the generic components unchanged. The consequence is, at best, a period of uncertainty and, at worst, potentially a very stressful situation [6]. This can be particularly challenging if they are unfamiliar with, or have limited experience in, the new specialty and the conditions they are going to encounter and hence be expected to deal with often in a very compressed timeframe. The consequence is the potential for a negative effect on patient care and patient safety, and there is a broadly held consensus among consultants and senior doctors that changeover days, especially the August changeover day, colloquially known as “Black Wednesday,” is a problematic period [7,8].

Medicine has ceased to be defined by its major branches and has iteratively evolved with specialization and subsequently sub-specialization, which has improved patient care and objectively improved outcomes. As a consequence, however, there are a far greater number of specialties and subspecialties that junior doctors have to familiarize themselves with in order to care for patients safely and effectively. Additionally, the limited exposure received during medical school or foundation years may also contribute to their unfamiliarity with certain fields within medicine, and a range of factors contributing to the lack of familiarity among junior doctors in complex specialties, such as HPB, can be identified [9,10].

Secondly, there is a huge shortage of qualified general practitioners in the UK, and it is a widely accepted view that if General Practice fails, then the NHS fails [11]. This understanding was the main driver that resulted in the government mandating that 50% of medical graduates should enter General Practice training. Although medical graduates can’t be forced to enter General Practice specialty training, there is presently considerable emphasis on making their decision “by choice - not by chance,” as recommended by the report commissioned by the Health Education England and The Medical Schools Council partnership. To ensure that General Practice is as appealing a career as possible for medical school graduates, there has been a steady increase in the quantity of community teaching, an increase in the proportion of exam questions that are set in primary and community settings, and an increase in the proportion of academic GPs and students exposed to them. This restructuring of the undergraduate curriculum and the change in emphasis has, by necessity, had an impact on the time spent by medical students in the hospital setting and their experience dealing with patients in secondary and tertiary institutions.

Junior doctors play a crucial role in the NHS and are essential to the functionality of this system as they provide front-line care to patients and contribute to the development of new medical knowledge and practices. While the vast majority of them find patient care rewarding, 80% found their job stressful and affecting their well-being [12]. Junior doctors joining the NHS face significant challenges, including long and unpredictable working hours, high workloads, limited support, lack of experience, difficulty maintaining a work-life balance, and navigating the complexities of the NHS [13,14]. Colleague's support, effective and supportive leadership, access to appropriate professional care, and a supportive learning environment can help mitigate the negative impact of working conditions and cultures experienced by many junior doctors [15,16].

Our experience in implementing an HPB-specific induction program has demonstrated substantial enhancements in junior doctors' knowledge and experience, a trend also observed in other units adopting specialty-specific induction programs [17-19]. These findings underscore the vital importance and effectiveness of structured, specialty-specific induction initiatives. However, it is essential to acknowledge the limitations of our study. Firstly, our research focused solely on the HPB specialty, potentially limiting its generalizability to other medical specialties. Additionally, the study's findings are drawn from a single institution's experience, and variations in resources, infrastructure, and teaching approaches across different healthcare facilities could impact the outcomes of specialty-specific induction programs. Furthermore, our study primarily assessed knowledge and experience improvements and did not directly measure their impact on patient outcomes or the long-term career choices of junior doctors.

## Conclusions

In summary, our study primarily centered on improving the knowledge and experience of junior doctors through specialty-specific induction programs, such as our HPB initiative and similar efforts. While our primary focus was on enhancing doctors' knowledge, it is worth noting that these programs may also have the potential to contribute to improved patient care. Continued investment in these initiatives remains vital, as they can play a role in strengthening the healthcare workforce and potentially elevating both medical knowledge standards and aspects related to patient care.
